# Ag Nanoparticle-Incorporated Natural Rubber for Mechanical Energy Harvesting Application

**DOI:** 10.3390/molecules26092720

**Published:** 2021-05-06

**Authors:** Pawanrat Suphasorn, Intuorn Appamato, Viyada Harnchana, Prasit Thongbai, Chalathorn Chanthad, Chomsri Siriwong, Vittaya Amornkitbamrung

**Affiliations:** 1 Materials Science and Nanotechnology Program, Faculty of Science, Khon Kaen University, Khon Kaen 40002, Thailand; spawanrat@kkumail.com (P.S.); Ap.intuorn@kkumail.com (I.A.); 2 Department of Physics, Khon Kaen University, Khon Kaen 40002, Thailand; pthongbai@kku.ac.th (P.T.); vittaya@kku.ac.th (V.A.); 3 Institute of Nanomaterials Research and Innovation for Energy (IN-RIE), NANOTEC-KKU RNN on Nanomaterials Research and Innovation for Energy, Khon Kaen University, Khon Kaen 40002, Thailand; 4 National Nanotechnology Center (NANOTEC), NSTDA, 111 Thailand Science Park, Paholyothin Road, KlongLuang, Pathum Thani 12120, Thailand; chalathorn@nanotec.or.th; 5 Materials Chemistry Research Center, Department of Chemistry and Center of Excellence for Innovation in Chemistry, Faculty of Science, Khon Kaen University, Khon Kaen 40002, Thailand; schoms@kku.ac.th

**Keywords:** natural rubber, Ag nanoparticles, dielectric constant, energy harvesting

## Abstract

The energy conversion performance of the triboelectric nanogenerator (TENG) is a function of triboelectric charges which depend on the intrinsic properties of materials to hold charges or the dielectric properties of triboelectric materials. In this work, Ag nanoparticles were synthesized and used to incorporate into natural rubber (NR) in order to enhance the dielectric constant for enhancing the electrical output of TENG. It was found that the size of Ag nanoparticles was reduced with the increasing CTAB concentration. Furthermore, the CTAB surfactant helped the dispersion of metallic Ag nanoparticles in the NR-insulating matrix, which promoted interfacial polarization that affected the dielectric properties of the NR composite. Ag nanoparticle-incorporated NR films exhibited an improved dielectric constant of up to almost 40% and an enhanced TENG performance that generated the highest power density of 262.4 mW/m^2^.

## 1. Introduction

Mechanical energy is among the most abundant forms of energy in our living environment. Mechanical energy exists in many forms, including all kinds of movement and vibration. Scavenging these ubiquitous energy forms is a crucial challenge for the development of green and sustainable renewable energy sources. A triboelectric nanogenerator (TENG) has been developed to harvest mechanical energy into electricity with the combination effect of contact electrification and electrostatic induction [1,2]. TENG is gaining attention due to its high energy conversion efficiency with the highest reported power density of 500 W/m^2^ [3], simple fabrication with a diverse material choice, many operation modes that can harvest mechanical energy in various forms and low production cost [2,4].

Polymeric materials are typically employed to fabricate TENGs, such as polytetrafluoroethylene (PTFE) [5,6], polydimethylsiloxane (PDMS) [7,8], polyvinylidene fluoride (PVDF) [9], polymethyl methacrylate (PMMA) [10] and polyimide (Kapton) [11]. Natural rubber (NR) or polyisoprene is among the raw materials used for manufacturing a wide variety of industrial products [12,13]. Most NR products involve direct contact with mechanical energy sources, and NR is among the triboelectric materials located in the triboelectric series with slightly negative polarity [14]. Therefore, the modification of NR for TENG application would be important to enhance the energy conversion performance of the TENG.

The energy conversion performance of TENG is a function of triboelectric charges which depend on the surface area and intrinsic properties of materials to hold charges or the dielectric properties of triboelectric materials [15,16]. The dielectric properties of triboelectric layers have been reported to significantly influence TENG performance [15,16,17,18,19,20,21]. The use of conductive fillers is an effective strategy to enhance the dielectric properties of polymer composite materials. Dispersing conductive fillers in an insulating polymer layer gives rise to the formation of micro-capacitors or the accumulation of charge in the local environment, which can enhance the dielectric properties of the composite by an interfacial polarization mechanism [22]. These conductive fillers include carbon nanotubes [23], Au [24], Ag [25], Al [26] and Ni nanoparticles [27]. The Ag nanoparticle (AgNP) is among the conductive fillers that are widely used for enhancing dielectric constant in polymer composites owing to many interesting properties, including good electrical conductivity, ease of fabrication and cost effectiveness [28,29,30,31]. Although there were many studies on the incorporation of AgNPs into NR, most of them were for biomedical and antibacterial applications [32,33,34,35,36]. The potential applications of pressure sensors in sportswear and wearable electronic were also proposed [37]. However, the use of the NR-Ag composite in TENG application for mechanical energy harvesting has not been reported to date.

In this work, AgNP-incorporated NR films were synthesized and used to fabricate triboelectric nanogenerators for the first time. NR films were filled with AgNPs in order to improve the energy conversion of NR-based TENG. AgNP-filled NR films exhibited an enhanced dielectric constant, which resulted in increased triboelectric charges and hence TENG electrical outputs. According to the enhancement of dielectric properties by the interfacial polarization mechanism, highly dispersed metal/semiconductor nanoparticles in the insulator matrix are required. AgNPs were synthesized by a chemical reduction method of AgNO_3_ using NaBH_4_ as a reducing agent and cetyltrimethylammonium bromide (CTAB) as a surfactant. The effects of surfactant concentration on the morphology of synthesized particles and dielectric properties of the NR composite films and TENG electrical output were investigated.

## 2. Results and Discussion

### 2.1. Ag Nanoparticles

**Figure 1:**
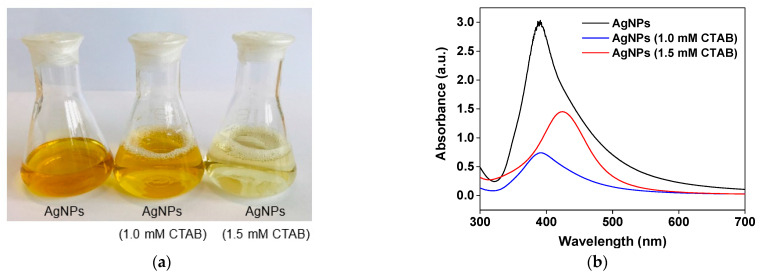
(**a**) Digital photographs of the solutions of Ag nanoparticles synthesized without the use of CTAB, with 1.0 mM CTAB and 1.5 mM CTAB. (**b**) UV–vis spectra of the AgNPs (1.0 mM CTAB), AgNPs (1.5 mM CTAB) and AgNPs without CTAB.

The aqueous solutions of AgNPs prepared at different conditions, including AgNPs without CTAB (without the use of surfactant), AgNPs (1.0 mM CTAB) and AgNPs (1.5 mM CTAB), exhibited various yellowish color shades, as presented in Figure 1a. UV–Vis absorption spectra of all the synthesized AgNPs are shown in Figure 1b. The UV–vis absorption wavelength of synthesized AgNPs without CTAB and AgNPs (1.0 mM CTAB) were in a similar range, but that of AgNPs (1.5 mM CTAB) exhibited a redshift toward a higher wavelength, suggesting a larger particle size.

**Figure 2:**
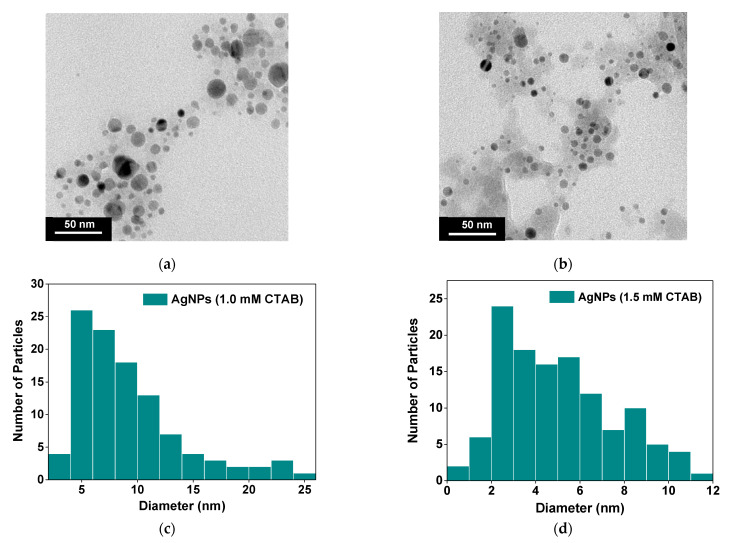
TEM image of (**a**) AgNPs (1.0 mM CTAB) and (**b**) AgNPs (1.5 mM CTAB). (**c**) and (**d**) are their particle size distribution analysis, respectively.

TEM images of the AgNPs (1.0 mM CTAB) and AgNPs (1.5 mM CTAB) are presented in Figure 2a,b, respectively, and their particle size distribution is displayed in Figure 2c,d, respectively. It was found that particle size analysis from the TEM images showed an inconsistency with the UV–vis absorption result in that the average particle size of the AgNPs (1.5 mM CTAB) was 5 nm, which was smaller than that of the AgNPs (1.0 mM CTAB), which was 9 nm. This can be ascribed to the increasing surfactant concentration causing the reduction in surface tension and stabilizing newly developed surfaces during homogenization and produce smaller particles [38,39]. This also resulted in the formation of a smaller number of particles (lower AgNP concentration), which can be observed from the lighter solution color at high CTAB concentration (Figure 1a). Therefore, the redshift observed in the UV–vis spectrum of the AgNPs (1.5 mM CTAB) could be attributed to the size of CTAB clusters, as observed in the TEM image in Figure 2b. The effect of particle size on the dielectric properties and TENG performance is discussed in Section 2.4.

### 2.2. NR-Ag Nanoparticle Composited Films

**Figure 3:**
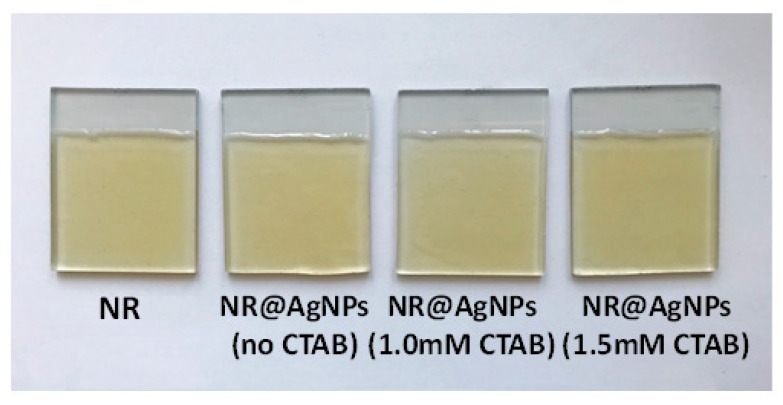
Digital photographs of the NR, NR@AgNP (no CTAB), NR@AgNP (1.0 mM CTAB) and NR@AgNP (1.5 mM CTAB) films cast onto ITO glass substrates as triboelectric electrodes.

All of these synthesized AgNPs were incorporated into NR latex samples, which were then coated on conductive ITO glass substrates (Figure 3) and subsequently used to assemble a TENG for electrical measurements.

**Figure 4:**
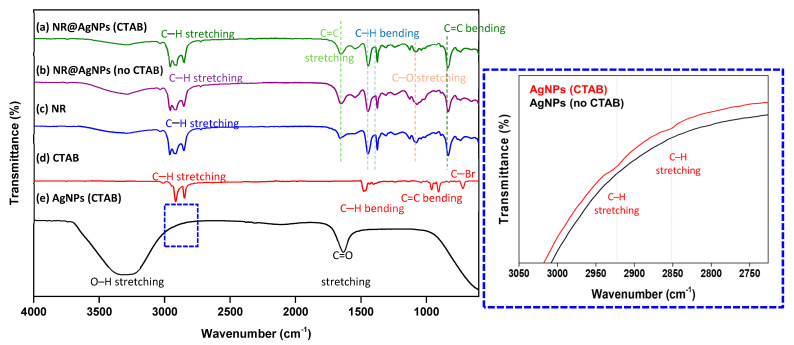
FTIR spectra of NR@AgNPs (CTAB) compared to NR@AgNPs (no CTAB), NR, pure CTAB and AgNPs and the inset presenting the presence of CTAB in the AgNPs (CTAB) but not in AgNPs (no CTAB).

The chemical structures of NR@AgNP composite films were investigated by a Fourier transform infrared spectroscopy (FTIR) analysis, as presented in Figure 4. FTIR spectra of the NR@AgNPs (no CTAB) and NR@AgNPs (CTAB) were compared to those of NR, CTAB and the synthesized AgNPs (with and without the use of CTAB). It was found that both FTIR spectra of NR@AgNPs (no CTAB) and NR@AgNPs (CTAB) were similar to that of pristine NR, which agreed well with the characteristic FTIR spectrum reported in the literature [40,41] corresponding to that of the cis-isoprene structure with the C–H stretching at 2960 cm^−1^, the symmetric and asymmetric stretching of the CH_3_ group at 2920 and 2855 cm^−1^ and the symmetric and asymmetric bending of the same group in the region of 1447 and 1376 cm^−1^. The bands at 3300–3400 cm^−1^ should be related to the N–H stretching, since the NRL was conserved in ammonia.

This indicated that AgNPs did not form bonding to NR, and hence, no change in the chemical structures of NR occurred. The AgNPs synthesized with and without CTAB showed almost identical FTIR spectra. The wide band at 3300–3400 cm^−1^ was caused by the stretching vibration of hydroxyl (O–H) groups; the 1637 cm^−1^ corresponds to the C=O stretching mode [42], and this result was consistent with other previous reports [43]. The difference was that the weak absorption bands at 2850 and 2920 cm^−1^ of C–H stretching from CTAB were observed in AgNPs (CTAB) but not in AgNPs (no CTAB), as revealed in the inset in Figure 4. However, the absorption characteristics of AgNPs were not detected in the NR@AgNPs (CTAB) due to the small volume fraction of AgNPs incorporated into NR (10%vol) (see Section 3.2).

### 2.3. TENG Electrical Outputs

**Figure 5:**
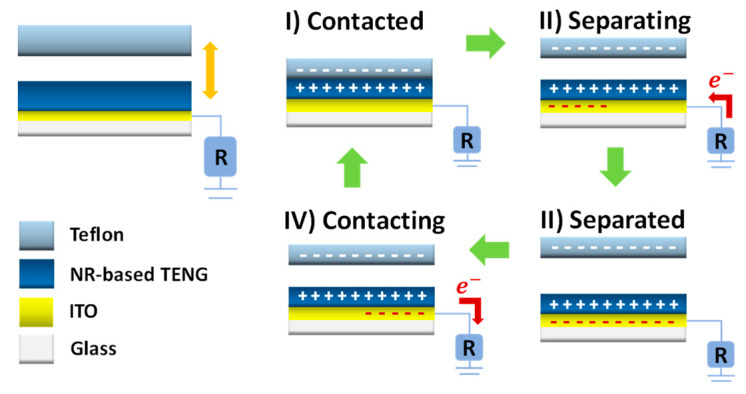
Schematic diagram of the fabricated TENG with working mechanism under a single electrode mode.

The TENG device configuration and its working mechanism under a single electrode mode are illustrated in schematic diagram in Figure 5. A Teflon sheet, which is the most negative triboelectric polarity, was employed as a contact triboelectric material. When the surfaces of the NR@AgNP composite film and Teflon are in contact, triboelectric charges with different signs are formed on the two surfaces upon electrification. When the two surfaces are separated, the potential decreases across the two surfaces, which can cause the free electrons to flow from ground to conductive electric to balance the created potential producing positive current signal. When the surfaces are brought into contact again, the potential reduces, and electrons flow back to the ground, generating a negative current signal.

**Figure 6:**
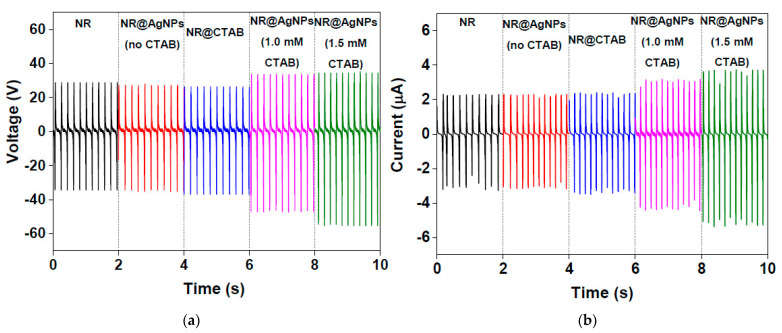
Electrical outputs: (**a**) voltage and (**b**) current of NR, NR@AgNP (without CTAB), NR@CTAB (no Ag), NR@AgNP (1.0 mM CTAB) and NR@AgNP (1.5 mM CTAB) TENGs.

The electrical voltage and current of NR, NR@AgNP (without CTAB), NR@CTAB (no Ag), NR@AgNP (1.0 mM CTAB) and NR@AgNP (1.5 mM CTAB) TENGs, measured under a single electrode mode at mechanical agitation frequency of 5 Hz, are presented in Figure 6a,b, respectively, and their values are listed in Table 1. It was found that adding Ag nanoparticles in the NR film improved the output voltage and current of the TENG. The NR@AgNP (1.5 mM CTAB) TENG generated the highest output voltage and current of 90.4 V and 9 µA, which were higher than NR@AgNPs (1.0 mM CTAB), NR@AgNPs (without CTAB), and two times larger than the pristine NR TENG. On the other hand, the outputs of the NR and NR@AgNP (without CTAB) TENGs were not different, suggesting that the CTAB surfactant influenced TENG performance. However, the presence of CTAB alone did not give rise to an improvement in TENG performance, as seen in the NR@CTAB (without Ag) TENG.

### 2.4. Dielectric Constant of the NR@AgNP Composite Materials

**Figure 7:**
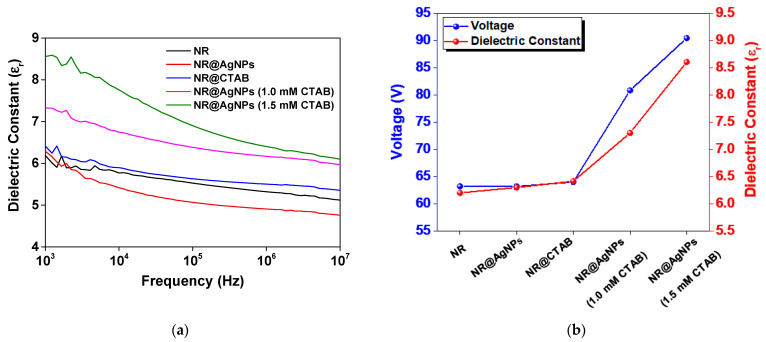
(**a**) Dielectric constant at various frequency ranges measured at room temperature and (**b**) the plot of TENG output voltage and dielectric constants of NR, NR@AgNP (without CTAB), NR@AgNP (1.0 mM CTAB) and NR@AgNP (1.5 mM CTAB) materials.

Dielectric properties of NR, NR@CTAB and all the NR@AgNP composite films were investigated, and their dielectric constants (ɛ_r_) at various frequency range are presented in Figure 7. It was found that adding AgNPs prepared with CTAB surfactant improved the dielectric properties of the NR composite. The dielectric constant of all the samples at 1 kHz is listed in Table 1. The NR@AgNPs (1.5 mM CTAB) had the highest dielectric constant, which was higher than those of the NR@AgNPs (1.0 mM CTAB), NR@CTAB, NR@AgNPs and pristine NR films, respectively. The higher dielectric constant of the NR@AgNPs (1.5 mM CTAB) compared to the NR@AgNPs (1.0 mM CTAB) was attributed to the smaller Ag particle size, which provided increased interfacial area and shorter interparticle distance [44].

According to the result above, the NR@AgNPs (1.5 mM CTAB) exhibited an improved dielectric constant and enhanced electrical output. However, the AgNPs without the use of CTAB did not show an improvement in dielectric the properties of the NR composite. This can be described by the fact that the NR molecule is nonpolar, with naturally negative surface charges due to nonrubber components, as well as the bare AgNPs that have negative surface charges. Moreover, AgNPs synthesized without surfactant generally experience the agglomeration of particles. Thus, the interfacial polarization mechanism in this case was relatively low. CTAB is a cationic surfactant with a positively charged hydrophilic head. CTAB could then act as a filler material in NR that generated polarization, giving rise to the increased dielectric constant in the NR@CTAB. However, CTAB molecules are large compared to Ag nanoparticles. The dielectric constant of NR@CTAB was then slightly higher than that of NR and NR@AgNPs, but lower than that of the NR@AgNPs (CTAB). This suggested that the surfactant plays an important role for dispersing Ag nanoparticles in the NR matrix, which consequently allowed interfacial polarization contributing to the increased dielectric constants in the NR@AgNPs (1.0 mM CTAB) and NR@AgNPs (1.5 mM CTAB).

It was found that the TENG electrical output is a function of the dielectric constant, as illustrated by the plot of TENG output voltage and dielectric constant of all the fabricated samples in Figure 7b. The maximum output voltage of TENG under the open circuit condition (V_oc_) is a function of triboelectric charge density (σ), as given by the expression in Equation (1) below [15].

(1) $$V_{oc}=\frac{\sigma x\left(t\right)}{\varepsilon_{0}}$$

where *x (t)* is the distance between the two surfaces of triboelectric material, and ɛ_o_ is vacuum permittivity. Apart from the two types of triboelectric materials and contact area that contribute to the triboelectric charge formation, the dielectric property is another crucial parameter affecting the output performance of TENG, since it indicates the charge holding ability of materials which can effectively intensify triboelectric charges.

### 2.5. Optimum Working Condition

**Figure 8:**
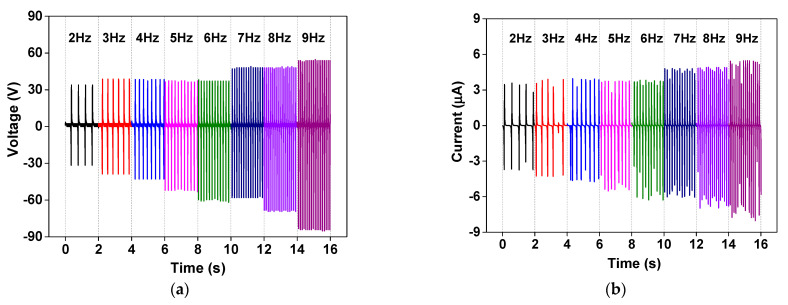
(**a**) Voltage and (**b**) current outputs of the NR@AgNPs (1.5 mM CTAB) TENG at various operation frequencies ranging from 2 to 9 Hz.

The dependence of electrical output on the working frequency of the fabricated TENG was also studied by measuring the electrical outputs of the NR@AgNP (1.5 mM CTAB) TENG at a frequency ranging from 2 to 9 Hz, which are presented in Figure 8. It was found that the output voltage and current increased with increasing frequencies and reached the maximum of 140 V and 13.5 µA at 9 Hz, respectively. The increased electrical outputs of the TENG at high frequencies were attributed to the charge accumulation due to very short contact cycles.

**Figure 9:**
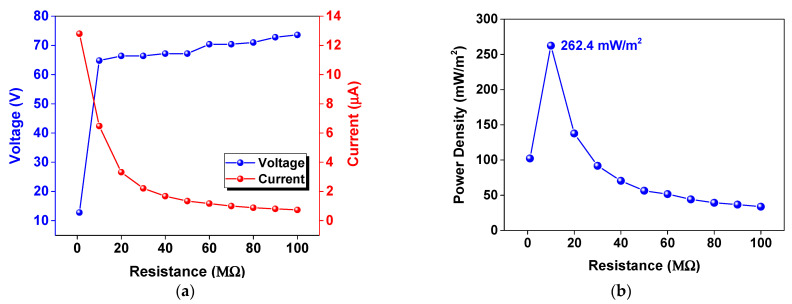
(**a**)Voltage and current outputs of the NR@AgNP (1.5 mM CTAB) TENG when connected to various external load resistances from 1 to 100 MΩ. (**b**) The corresponding power densities derived from (**a**).

The delivered power density of the TENG was investigated by connecting to various load resistances ranging from 1 to 100 MΩ. The dependence of output voltage and current of the NR@AgNP (1.5 mM CTAB) TENG on external load resistances and the corresponding power density is presented in Figure 9a,b, respectively. The highest delivered power density of 262.4 mW/m^2^ was achieved at a matched load resistance of 10 MΩ, as shown in Figure 9b. The power density was found to decrease with increasing load resistances.

**Figure 10:**
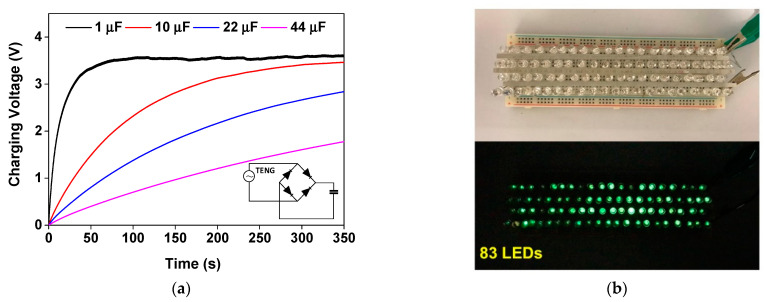
(**a**) Voltage profile of the NR@AgNP (1.5 mM CTAB) TENG to charge 1, 10, 22 and 47 µF; (**b**) the demonstration of lighting up 83 green LEDs connected in series by generated electrical power from the fabricated TENG.

This delivered electrical power was sufficient to charge 1, 10, 22 and 47 µF capacitors, as presented in the charging profile in Figure 10a. The fabricated TENG can charge a 1 µF capacitor to 3.5 V in 60 s and a 47 µF capacitor to 1.5 V in 360 s. Moreover, the generated electricity was demonstrated to light up 83 green LEDs connected in series (Figure 10b).

The fabricated NR@AgNP TENG shows the energy production performance approaching the propeller PTFE-based TENG with a power density of 283.95 mW/m^2^ [45]; a higher than a triple cantilever-based triboelectric nanogenerator made from PDMS and ZnO triboelectric layers with a power density of 252.3 mW/m^2^ [46]; and the flexible triboelectric generators based on patterned conductive textile and PDMS layers with a power density of 181.9 mW/m^2^ [47].

## 3. Materials and Methods

### 3.1. Synthesis of Ag Nanoparticles

Silver nitrate (AgNO_3_, RCI Labscan, 99.8% purity) was used as the starting material for the preparation of AgNPs. The AgNPs were prepared by a chemical reduction method based on the method proposed in references [48,49]. Sodium borohydride (NaBH_4_, Qrec, 96%) was used as a reducing agent and CTAB (Ajax Finechem, 98%) as a surfactant. All the solutions were prepared in deionized (DI) water. An amount of 60 mL of 1.0 mM NaBH_4_ was prepared and kept in an ice bath. The 60 mL of 1 mM AgNO_3_ was added to 50 mL of CTAB solutions at 1.0 and 1.5 mM. Then, AgNO_3_ solution was added dropwise to NaBH_4_ solution. The reaction mixture was stirred vigorously on a magnetic stirrer for 40 min until the color of the solution changed to yellow. The excess CTAB was removed by centrifugation and supernatant removal. The AgNPs prepared with 1.0 and 1.5 mM CTAB are called AgNPs (1.0 mM CTAB) and AgNPs (1.5 mM CTAB), respectively.

### 3.2. Preparation of NR-AgNP Composite Films

The commercial high-ammonia natural rubber latex (NRL) with dry rubber content of 61% used in this work was purchased from the Thai Rubber Latex Group Public Co., Ltd., Thailand. The NRL was stirred for 2 h to partially remove ammonia. Then, the NRL was mixed with the AgNP solution at NRL/AgNP ratios of 1 mL:100 µL. The mixtures were stirred for 10 min to obtain a homogeneous mixed solution. The mixed NRL/AgNP solution was immediately casted on a 4 × 4 cm^2^ ITO glass substrate. The thicknesses of the films were controlled by fixing an NRL/AgNP suspension at 2 mL per sample. Three samples were prepared for each experimental condition. Then, the samples were allowed to naturally dry at room temperature for 4 days. Finally, the dried films were further heated at 80 °C for 2 h.

### 3.3. Material Characterization

The synthesized Ag particles were analyzed by a UV–Vis spectrometer (UV-1800, Shimadzu) at a range of visible lights of 300–700 nm and a transmission electron microscope (TEM; TECNAI, G220) to study particle morphologies and their size distributions. Dielectric properties were probed using a Keysight E4990A impedance analyzer in a frequency range from 10^3^ to 10^8^ Hz at room temperature. Chemical functional group analysis was performed on the 1 × 1 cm^2^ NR composite films using an FTIR spectroscope (TENSOR27).

### 3.4. TENG Electrical Output Measurement

A single electrode vertical contact-separation configuration was used for the performance testing of TENGs. The set-up TENG device consisted of the NR composite as a bottom triboelectric electrode and PTFE as a top triboelectric electrode. The voltage and current outputs were monitored using an oscilloscope (Tektronix DPO2002B) and a digital ammeter (Kiethley DMM6500), respectively.

## 4. Conclusions

The NR-Ag nanoparticle composites were fabricated and used to fabricate TENGs to harvest mechanical energy. The addition of Ag nanoparticles, which were synthesized by a chemical reduction method with the use of CTAB surfactant, was found to improve the dielectric constant of NR materials. It was found that the AgNP size reduced at increasing CTAB concentration. AgNPs synthesized at CTAB 1.5 mM CTAB were well dispersed with a smaller particle size that that of the 1.0 mM CTAB. NR@AgNP (1.5 mM CTAB) TENG exhibited the highest dielectric constant due to the enhanced interfacial polarization of the incorporation of well-dispersed AgNPs in NR resulting in the enhancement of the electrical output of the fabricated TENG that generated the highest electrical output with the highest power density of 262.4 mW/m^2^.

## Figures and Tables

**Table 1:** Electrical output voltage and current of NR, NR@AgNP (without CTAB), NR@AgNP (1.0 mM CTAB) and NR@AgNP (1.5 mM CTAB) TENGs and their dielectric constants **(ɛ_r_)** at 1 kHz.

Samples	TENG Output		Dielectric Constant (ɛ_r_)
	Voltage (V)	Current (µA)	
NR	63.2	5.6	6.2
NR@AgNPs	63.2	5.5	6.3
NR@CTAB	64.0	5.9	6.4
NR@AgNPs (1.0 mM CTAB)	80.8	7.6	7.3
NR@AgNPs (1.5 mM CTAB)	90.4	9.1	8.6

## Data Availability

The data presented in this study are available on request from the corresponding author.

## Sample Availability

The samples of NR-Ag composites are available from authors upon the reasonable request.
